# Publication Trends Over 55 Years of Behavioral Genetic Research

**DOI:** 10.1007/s10519-016-9786-2

**Published:** 2016-03-18

**Authors:** Ziada Ayorech, Saskia Selzam, Emily Smith-Woolley, Valerie S. Knopik, Jenae M. Neiderhiser, John C. DeFries, Robert Plomin

**Affiliations:** 1 MRC Social, Genetic and Developmental Psychiatry Centre, Institute of Psychiatry, Psychology & Neuroscience, King’s College London, DeCrespigny Park, London, SE5 8AF UK; 2 Division of Behavioral Genetics, Rhode Island Hospital, Providence, RI USA; 3 Department of Psychiatry & Human Behavior, Warren Alpert Medical School, Brown University, Providence, RI USA; 4 Department of Psychology, The Pennsylvania State University, State College, PA USA; 5 Institute for Behavioral Genetics, University of Colorado, Boulder, CO USA

**Keywords:** Behavioral genetics, Database, Quantitative genetics, Molecular genetics

## Abstract

We document the growth in published papers on behavioral genetics for 5-year intervals from 1960 through 2014. We used 1861 papers published in *Behavior Genetics* to train our search strategy which, when applied to Ovid *PsychINFO,* selected more than 45,000 publications. Five trends stand out: (1) the number of behavioral genetic publications has grown enormously; nearly 20,000 papers were published in 2010–2014. (2) The number of human quantitative genetic (QG) publications (e.g., twin and adoption studies) has steadily increased with more than 3000 papers published in 2010–2014. (3) The number of human molecular genetic (MG) publications increased substantially from about 2000 in 2000–2004 to 5000 in 2005–2009 to 9000 in 2010–2014. (4) Nonhuman publications yielded similar trends. (5) Although there has been exponential growth in MG publications, both human and nonhuman QG publications continue to grow. A searchable resource of this corpus of behavioral genetic papers is freely available online at http://www.teds.ac.uk/public_datasets.html and will be updated annually.

**Electronic supplementary material:**

The online version of this article (doi:10.1007/s10519-016-9786-2) contains supplementary material, which is available to authorized users.

## Introduction

Although the origins of behavioral genetic research trace back to Francis Galton in the 19th century (Galton 1869), the modern era can be conveniently marked with the field-defining book, *Behavior Genetics* by Fuller and Thompson in 1960. The growth of behavioral genetic research since then is accelerating as the use of genetically sensitive designs such as twin and adoption studies permeates all domains of behavioral science (Knopik et al. in press). The rapid advances in research using DNA itself seems to have fueled even greater growth during the past decade.

The purpose of this paper is to document the growth of behavioral genetic research publications since 1960 and to create a searchable resource. We compare growth in quantitative genetic (QG) and molecular genetic (MG) human and nonhuman research. Our search for scientific publications on behavioral genetics found over 45,000 publications from 1960 through 2014 on which we based these analyses. We have compiled these references (authors, title, journal, year) with their abstracts, key words and PubMed ID/DOI numbers in an online resource, (the *Behavioral Genetics index*, BGi), that is freely available and searchable and which will be updated on an annual basis: http://www.teds.ac.uk/public_datasets.html.

## Method

Our goal was to identify all published papers between 1960 and 2014 on the genetics of human and non-human behavior, including both quantitative and MG studies. We searched for publications using the search engine Ovid, selecting the most up-to-date version of the database *PsychINFO*. *PsychINFO* includes more than four million records from more than 2500 journals that publish papers related to behavior. We limited our search to *PsychINFO* to make the task more manageable, although we admit that *PsychINFO* misses some behavioral genetic papers among the 1 billion records including an additional 10,000 journals, for example, covered by the *Science Citation Index.*

In our search, we specified *journal article* or *review* only. We excluded conference presentations for two reasons: they rarely include abstracts, which would make them difficult to categorize, and they are often later published as papers, which would result in duplication.

Definitional issues and search criteria complicate the search for published papers on behavioral genetics. Definitional issues begin with the basic questions ‘what is behavior?’ and ‘what is genetics?’. We are confident that the bulk of papers that we selected would meet anyone’s definition of behavioral genetics—for example, twin studies or DNA association studies of psychopathology, personality and cognitive abilities and disabilities. However, problems emerge at the borders. Our aim was to be inclusive within reasonable bounds in order to capture most behavioral genetic publications. For example, if behavior is defined as observable activity of an organism rather than a particular organ, then what about neuroimaging studies of brain activity? We included these papers, although we attempted to exclude publications that refer to the ‘behavior’ of cells or molecules. We also excluded papers that looked at methodological issues without specifying a phenotype. The word *genetic* is especially far-reaching, but we focused on the inheritance of individual differences within a species, excluding, for example, research on average genetic differences between species.

It is also difficult to avoid arbitrariness in selecting search criteria to identify papers that fall within these broad definitional boundaries of behavioral genetics. We avoided specific phenotypes in our search terms so as not to limit the scope of papers captured, but instead focused on methodological keywords which are common to behavioral genetics as a field. In our online resource, we include the actual search terms used, which will facilitate the use of different criteria. We expect that few will object to the papers we have selected, although some might wish to cast a wider net.

We avoided some of these definitional and search criteria issues by ‘training’ our search strategy on the 2107 papers in the journal *Behavior Genetics* from 1960 to 2014 (for a flow diagram of the search process, see Supplementary Figure S1). Following our inclusion criteria above, we omitted 246 papers, resulting in a total of 1861 publications. By using the journal *Behavior Genetics*, we make the assumption that papers published in this journal define research on behavioral genetics. We began by identifying a list of commonly used keywords which enabled us to classify the papers into four categories: human, non-human, QG (e.g., twin and adoption studies) and MG (e.g., linkage and association studies). We also identified those papers that fell into more than one category. We refined the search strategies for these four categories until we could no longer improve the results. (The final search codes are listed in Supplementary Methods S1). We then applied these search terms to publications in *Behavior Genetics*. Although *Behavior Genetics* was the journal on which our search terms were trained, we conducted this analysis for three reasons. First, this test indicates the best possible results that could be expected when we applied these search terms to other journals. Second, the 1861 papers in *Behavior Genetics* were a manageable number for manual coding. Third, we could determine false negative rates because all the ‘correct’ papers had been identified.

We then applied these search strategies to the *PsychINFO* database. The arbitrariness of definitions and search terms and the inclusion of false positives is not likely to jeopardize our results documenting the growth of research in behavioral genetics because the same criteria were applied across all years.

## Results

### Training search criteria on the journal *Behavior Genetics*

The results for our training of search criteria using the *Behavior Genetics* journal are presented in Table 1. When combining search codes for all categories we recovered 83 % of the 1861 papers published in *Behavior Genetics* (see Table 1). Our individual codes for the four categories recovered the following percentage of papers: 84 % human quantitative, 78 % human molecular, 76 % non-human quantitative, and 82 % non-human molecular. Based on our coding, the percentage of papers that were incorrectly identified in the four categories (false positives) were 4, 17, 8, and 14 %, respectively (see Table 1). False positives primarily consisted of miscategorizations, for example, when a QG paper was classified as a MG paper. Publications that were not picked up by our code—for example, papers in which our keywords did not appear in the title or abstract—were classified as false negatives.

**Table 1:** Keyword training descriptive statistics

	BG database	Ovid results	Overlap	Overlap %	FP %	FN %
Human QG	738	649	619	83.9	4.1	16.1
Human MG	206	194	160	77.7	16.5	22.3
Nonhuman QG	605	497	457	75.5	8.2	24.5
Nonhuman MG	337	319	276	81.9	13.5	18.1
Codes combined^a^	1861	1542	1537	82.6	<.01	17.5

*BG database* number of publications in the journal *Behavior Genetics* for each category, *Ovid result* number of publications yielded by the search scripts, *Overlap %* the degree of overlap achieved, *FP* false positives and *FN* false negatives

^a^Codes combined is less than the sum of each category because we have captured only unique publications by using the Boolean operator ‘OR’

In Table 1, the sum of publications from individual codes (1886) exceeded the total number of papers in our training database (1861). This discrepancy occurs because 1 % of papers were correctly classified into more than one category, for example, a quantitative family study that also used linkage analysis.

### Applying the search criteria to *PsychINFO*

Our search criteria, trained on *Behavior Genetics,* yielded 46,941 papers from 1917 journals including *Behavior Genetics* when applied to Ovid *PsychINFO*. Spot-checking 10 % of these publications indicated that, on average, 75 % of the search results were correctly selected and assigned to one of the four categories (see Table 2, which shows an average false positive rate of 25 %). Based on this spot-check, we can estimate that around 36,800 publications are ‘true’ behavioral genetics papers. Rather than attempting to refine these results manually, we chose to accept the false positives, which has the advantage of keeping the search completely objective and also facilitating regular updates.

**Table 2:** Number of total findings from *PsychINFO* database search

	Total results	FP %
Human QG	11,694	27
Human MG	18,296	21
Nonhuman QG	6501	23
Nonhuman MG	12,079	27

The average FP rate was 24.5 %

*FP* false positives

For our wider search in Ovid *PsychINFO*, the sum of publications (48,571) from individual codes exceeded the total number of papers captured when we combined the codes with the Boolean operator ‘OR’ (46,941). This indicated that 3.5 % of the identified papers fell into more than one category, which included papers that combine human and nonhuman research as well as QG and MG.

The discrepancy between the overlapping categories for the training database (1 %) compared to overlap identified in the wider search (3.5 %) is likely due to the varying methodological scope within the 1917 journals in the wider search. As our training database was only based on *Behavior Genetics*, other journals may be more likely to attract papers which would fall into two or more categories.

**Fig. 1:**
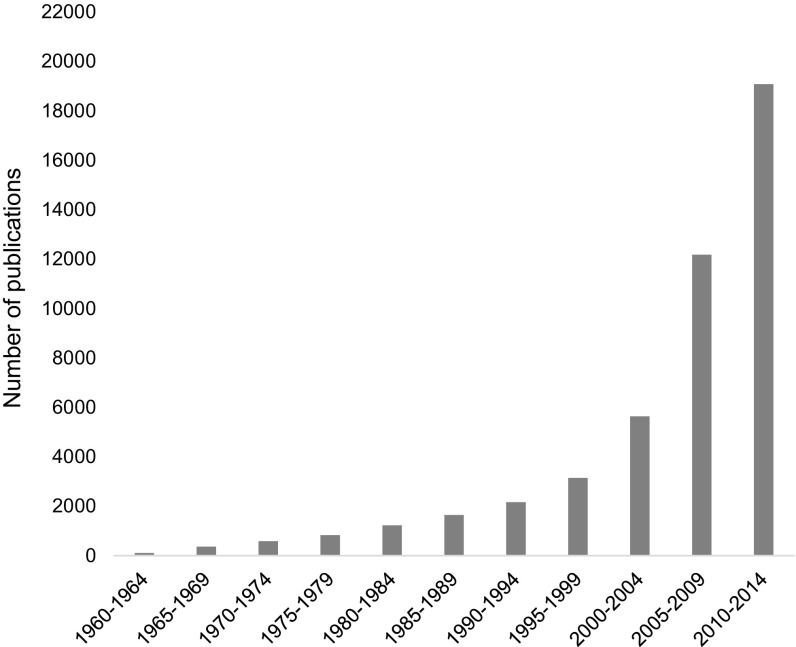
Total number of behavioral genetic papers published in 5-year intervals since 1960

Figure 1 shows the growth in publications by lustrum since 1960. After a slow start in the 1960s and 1970s, the number of publications has rapidly grown. From 1995 onwards, the number of publications has almost doubled every 5-year interval, with nearly 20,000 papers published during 2010–2014.

Although only 3 % of the 46,941 papers were published in *Behavior Genetics*, that percentage has decreased dramatically over the years (from 12 % in 1970–1989 to 4 % in 1990–2009 and to 1 % in 2010–2014) as other journals have increased their publication of behavioral genetic research. Most of those journals are in psychology (37 %) and psychiatry (12 %), but now include fields as diverse as education, economics, political science, and sociology.

**Fig. 2:**
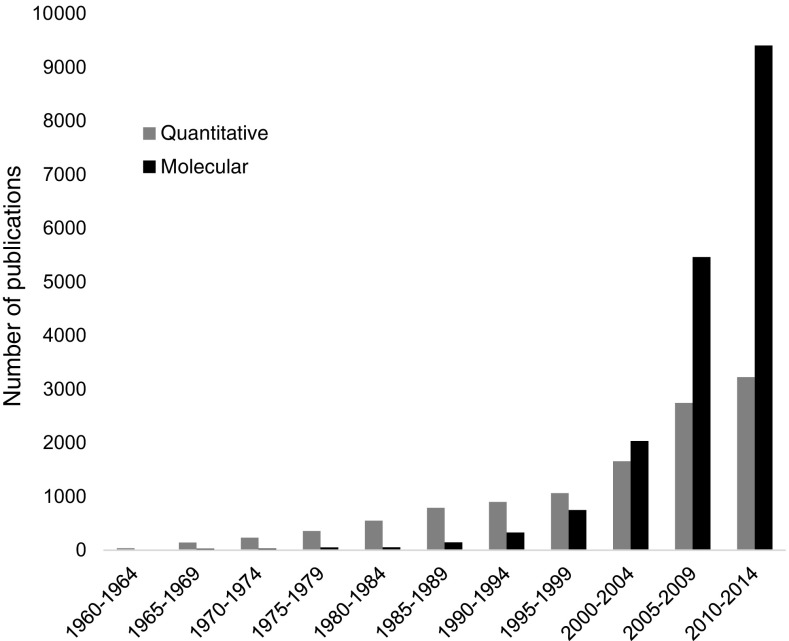
Number of human behavioral genetic papers by lustrum since 1960, comparing quantitative and molecular genetic research

Figure 2 compares the number of publications in human QG and MG research. The number of QG publications has increased steadily, with more than 3000 papers published in 2010–2014. The most striking result is the increase in MG papers: from 2034 in 2000–2004 to 5464 in 2005–2009 to 9406 in 2010–2014.

**Fig. 3:**
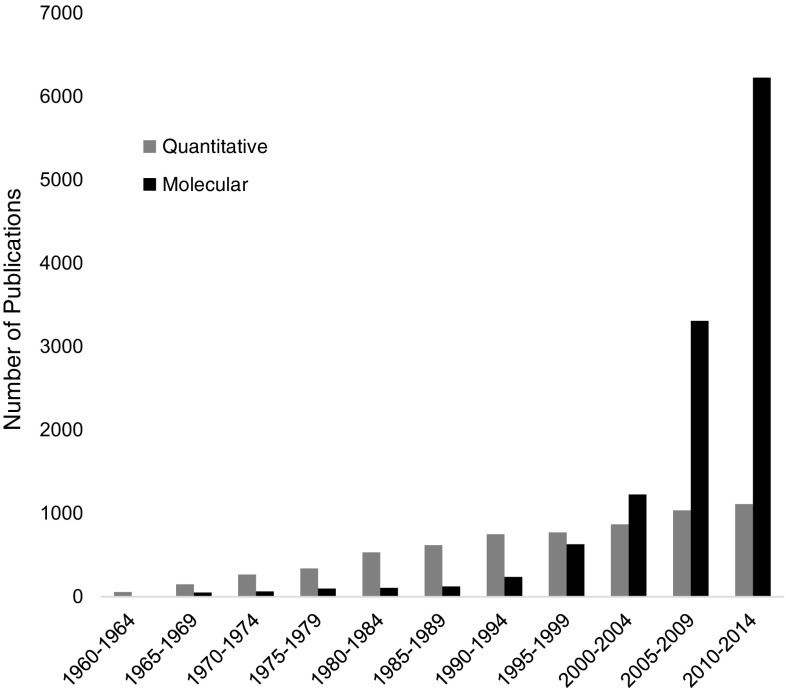
Number of nonhuman behavioral genetic papers published by lustrum since 1960, comparing quantitative and molecular genetic research

Figure 3 illustrates the number of nonhuman QG and MG publications. The number of QG publications has increased modestly since 1960, with 1111 QG papers published during 2010–2014. What is striking is the rapid increase in nonhuman MG publications during the last decade, from 1227 papers in 2000–2004 to 6227 papers in 2010–2014.

## Discussion

The growth of behavioral genetic publications signals a more balanced view in the behavioral sciences that recognizes the importance of genetic as well as environmental influences. It is also encouraging that behavioral genetic research has permeated most of the behavioral sciences. Only 3 % of the 46,941 publications were published in *Behavior Genetics* and this percentage has decreased from 12 % in the 1970s and 1980s to 1 % in 2010–2014. Behavioral genetic papers are increasingly being published in other journals, not just in psychology and psychiatry, but also in fields as diverse as education, economics, political science and sociology.

Although the growth of MG research in recent years is especially striking, QG research has also grown steadily. These two worlds of genetics are complementary. QG research reveals the net effect of genetic influence regardless of the number of genes involved, their effect sizes, or the complexity of their interactions. MG research attempts to identify these genes of small effect, which raises the problem of missing heritability (Manolio et al. 2009). Although fewer than 3 % of all papers combine QG and MG strategies in our wider search, this percentage seems certain to increase as researchers capitalize on the complementarity of quantitative and molecular genetics. Moreover, these two worlds of genetics are coming together with the availability of genome-wide polygenic scores that capture some of the heritability of behavioral traits and disorders (Plomin and Simpson 2013).

As noted earlier, a searchable resource of this corpus of behavioral genetic papers, the *Behavioral Genetics index* (BGi), has been compiled that includes references, abstracts, key words and PubMed ID/DOI numbers: http://www.teds.ac.uk/public_datasets.html. The BGi is freely accessible and searchable and will be updated annually. It also allows users to search by phenotype to identify publications trends and produce graphs depicting their trajectory over time. The search terms used to create this resource are included on the website; we welcome suggestions for improving the search.

Considering the striking finding that only 15 % of articles account for half of citations in the 1.8 million journal articles published each year (STM report, Ware and Mabe 2012), more attention needs to be given to searchability. Our work emphasizes three obvious suggestions for making behavioral genetic papers more searchable, which will also make papers more likely to be cited and thus to have greater impact. First, because key terms are central to searchability, they should be carefully chosen to specify phenotype, sample and method; distinctive characteristics of a paper that cannot be included in key words should be mentioned in the title or abstract because the main body of the journal article is not searched. Second, it helps an area if there is an agreed-upon name for it. For example, SNP-based heritability estimates have at least half a dozen different labels, which makes a search more difficult. In contrast, although there are many labels for polygenic scores, they nearly all contain the phrase *polygenic score*, which facilitates their search. Third, if key terms are composed of multiple words (e.g. “Family study”), these should appear adjacent to one another (e.g. “A family study of 300 siblings” instead of “300 siblings were studied in a family-based design”). Our online searchable resource can be used to test keywords proposed for a manuscript by entering these keywords to ensure that they select appropriate papers.

We have produced the largest comprehensive behavioral genetic database to date, documenting the field’s publications from 1960. However, we acknowledge that our results include some incorrectly identified papers as indicated by the false positive rate in our spot-checks. As false positives are likely to be evenly distributed over the 55-year interval, this should not affect the trends observed. Another potential limitation is that we trained our search terms on the journal *Behavior Genetics.* This may have introduced biases due to its methodological content, which is more heavily focused on complex behavior compared to some other journals. Potentially these biases may have increased the discrepancy between the false positive rates of our training search relative to the wider search in *PsychInfo*.

 Our online resource is of course limited to the quantity, not quality, of behavioral genetics papers. We acknowledge that the health of a field is judged more by the quality than the quantity of its research. However, we have argued elsewhere that the quality of research in behavioral genetics is also impressive (Plomin et al. 2016). In contrast to the current crisis of failures to replicate results (Open Science Collaboration 2015), behavioral genetic research has yielded robustly replicated findings that are large, both in terms of effect size and potential impact on science and society.

## Electronic supplementary material

Below is the link to the electronic supplementary material.

Supplementary material 1 (DOCX 74 kb)
