# Supplementary material for: Publication Trends Over 55 Years of Behavioral Genetic Research
Source: Behav Genet. 2016 Mar 18;46(5):603–7. doi: 10.1007/s10519-016-9786-2 (PMC5206393; doi:10.1007/s10519-016-9786-2)
Supplement: Supplementary file 1 — Supplementary material 1 (DOCX 74 kb) [file 10519_2016_9786_MOESM1_ESM.docx]

**Supplementary Material**

***Supplementary Figure***

Figure S1. Flow diagram of search process


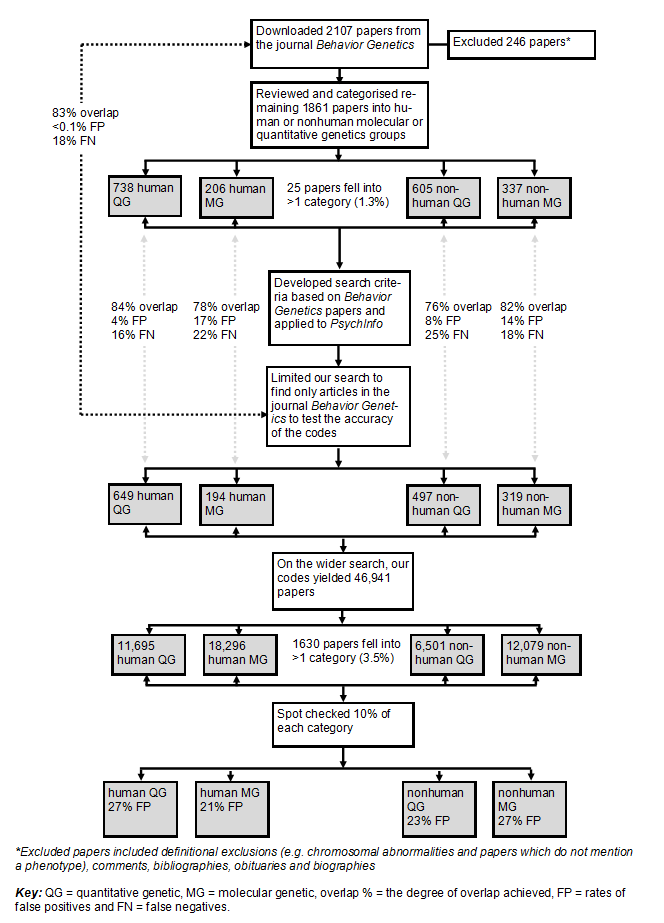


***Supplementary Methods S1***

Search Codes:

**Human Quantitative Genetics:**

*Twin studies:*

(Twin* AND (MZ OR DZ OR monozygotic OR dizygotic OR Heritabil* OR <individual differences> OR zygosity OR <shared environment> OR <common environment> OR <non-shared environment> OR <non shared environment> OR <nonshared environment> OR registry OR pairs OR Swedish OR Colorado OR <Early Development> OR Danish OR study OR concordance OR <reared apart> OR phenotyp* OR longitudinal OR <additive genetic> OR quantitative OR additive OR genetic OR bivariate OR multivariate OR univariate OR cholesky OR biometric))

*Family Studies:*

((((famil*) and (twin or spouse or (family adj stud*) or (assortative adj mating) or heritab* or resemblance or sib* or pair or parent offspring or phenotyp* or relative*1 or pedigree*1 or (twin*1 AND (sib* or spouse*1 or parent*1 or parental) or inbre* or X-link* or intelligence or mating))) AND (exp Behavioral Genetics/ OR exp Heritability/ OR exp Assortative Mating/ OR exp Genetics/ or exp Phenotypes/ or exp Individual Differences/ or exp Etiology/

or exp Twins/)) NOT (linkage or loci or mutation or marker*1 or SNP* or polymorphism or chromosom* or fragile or adoption or adopted or adoptive or "adoption (child)"/ or exp adopted children/ or exp adoptees/ or exp Human Sex Differences/))

*Adoption Studies:*

(((Adoption or adoptee*1 or adoptive) AND (CAP or families or twin*1 or heritab* OR heredit* or sib* or biologic*)) NOT (SNP* or candidate or polymorphism or marker*1))

*GCTA:*

((GCTA) AND (SNP* OR <single nucleotide polymorph>* OR heritability OR quantitative OR <complex-trait> OR <complex trait>))

*GPS:*

(<polygenic score> OR <genome wide scor*> OR <polygenic risk score> or <genome wide polygenic risk score> or <genome wide polygenic score>)

**Combined Human Quantitative Genetics:**

((Twin* AND (MZ OR DZ OR monozygotic OR dizygotic OR Heritabil* OR <individual differences> OR zygosity OR <shared environment> OR <common environment> OR <non-shared environment> OR <non shared environment> OR <nonshared environment> OR registry OR pairs OR Swedish OR Colorado OR <Early Development> OR Danish OR study OR concordance OR <reared apart> OR phenotyp* OR longitudinal OR <additive genetic> OR quantitative OR additive OR genetic OR bivariate OR multivariate OR univariate OR cholesky OR biometric)) OR ((((famil*) and (twin or spouse or (family adj stud*) or (assortative adj mating) or heritab* or resemblance or sib* or pair or parent offspring or phenotyp* or relative*1 or pedigree*1 or (twin*1 AND (sib* or spouse*1 or parent*1 or parental) or inbre* or X-link* or intelligence or mating))) AND (exp Behavioral Genetics/ OR exp Heritability/ OR exp Assortative Mating/ OR exp Genetics/ or exp Phenotypes/ or exp Individual Differences/ or exp Etiology/ or exp Twins/)) NOT (linkage or loci or mutation or marker*1 or SNP* or polymorphism or chromosom* or fragile or adoption or adopted or adoptive or "adoption (child)"/ or exp adopted children/ or exp adoptees/ or exp Human Sex Differences/)) OR (((Adoption or adoptee*1 or adoptive) AND (CAP or families or twin*1 or heritab* OR heredit* or sib* or biologic*)) NOT (SNP* or candidate or polymorphism or marker*1)) OR ((GCTA) AND (SNP* OR <single nucleotide polymorph>* OR heritability OR quantitative OR <complex-trait> OR <complex trait>)) OR (<polygenic score> OR <genome wide scor*> OR <polygenic risk score> or <genome wide polygenic risk score> or <genome wide polygenic score>))

**Human Molecular Genetics:**

*GWAS:*

(((GWAS OR GWA OR <genome wide association study> OR <genome wide association> OR <genome-wide association>) AND exp genome/) NOT (trisomy or down syndrome or turner or XXX or 22q11))

*Linkage:*

((((Linkage OR QTL) AND (analysis or QTL OR <quantitative trait loc*> OR chromosome*1 OR loci OR locus OR map* OR allel* OR SNP*1 or linkage)) AND (exp Genetic Linkage/ or exp genetics/ or exp genes/ or exp genome/ or exp Quantitative Trait Loci/ or exp Chromosomes/ or polymorphism/)) NOT (trisomy or down syndrome or turner or XXX or 22q11 or X ajd link* or X-link* or candidate))

*Association studies (incl candidate gene gene studies and GWAS):*

((((GWAS or GWA or (genome adj wide adj association) or (genome-wide adj association) or candidate or (candidate adj gene*) or polymorphi* or SNP*) AND (GWAS or gene* or loci OR <quantitative trait> OR chromosome OR polymorphism OR variant or SNP OR <single nucleotide polymorphism> OR variant* or associat* or genotype* or allel* or SNP* or marker*)) NOT (trisomy or down syndrome or turner or XXX or 22q11 or X ajd link* or X-link* or GCTA)) AND ( exp genome/ or exp polymorphism/ or exp genotypes/ or exp genes/ or exp genetics/ ))

*Epigenetics:*

((transcription or epigenetic* or (gene adj expression)) and (profiling or express* or methyl* or microarray or sequenc* or regulat* or transcript* or DNA or <deoxyribonucleic acid> or CAMP) NOT (trisomy or (down adj syndrome) or turner or XXX or 22q11 or sex-linked or x-link*))

**Combined Human Molecular Genetics:**

*(*(((GWAS OR GWA OR <genome wide association study> OR <genome wide association> OR <genome-wide association>) AND exp genome/) NOT (trisomy or down syndrome or turner or XXX or 22q11)) OR ((((Linkage OR QTL) AND (analysis or QTL OR <quantitative trait loc*> OR chromosome*1 OR loci OR locus OR map* OR allel* OR SNP*1 or linkage)) AND (exp Genetic Linkage/ or exp genetics/ or exp genes/ or exp genome/ or exp Quantitative Trait Loci/ or exp Chromosomes/ or polymorphism/)) NOT (trisomy or down syndrome or turner or XXX or 22q11 or X ajd link* or X-link* or candidate)) OR ((((GWAS or GWA or (genome adj wide adj association) or (genome-wide adj association) or candidate or (candidate adj gene*) or polymorphi* or SNP*) AND (GWAS or gene* or loci OR <quantitative trait> OR chromosome OR polymorphism OR variant or SNP OR <single nucleotide polymorphism> OR variant* or associat* or genotype* or allel* or SNP* or marker*)) NOT (trisomy or down syndrome or turner or XXX or 22q11 or X ajd link* or X-link* or GCTA)) AND ( exp genome/ or exp polymorphism/ or exp genotypes/ or exp genes/ or exp genetics/ )) OR ((transcription or epigenetic* or (gene adj expression)) and (profiling or express* or methyl* or microarray or sequenc* or regulat* or transcript* or DNA or <deoxyribonucleic acid> or CAMP) NOT (trisomy or (down adj syndrome) or turner or XXX or 22q11 or sex-linked or x-link*)))

**Animal Quantitative Genetics:**

((((inbred* or diallel or reciprocal hybrids or related strains or isofemale lines or inbreeding or reciprocal crosses or isogenic lines or isogenic strain* or hybrid mice).ab,ti.) OR ((selectively bred or bidirectionally selected or experimentally selected or bidirectional selection or divergent selection or cross breed or cross breeding or cross-breeding or artificial selection or high-selected or genetically selected or genetic selection or selective breeding or directional selection or F1 hybrid* or reciprocal F1 or selected lines or selection lines).ab,ti.) OR ((strain difference*).ab,ti. or Animal Strain Differences/) OR ((assortative mating).ab,ti) OR ((quantitative genetic or behavior* genetic or Behavior*-genetic or heritabilit* or heritable or genetic correlation or additive genetic).ab,ti.) OR (((genetic analysis) AND behaviour*).ab,ti.) OR ((drosophila AND (selected or strains or behaviour* or genetic)).ab,ti.)) NOT (mutant* or mutated or mutations or mutagenic or receptor or albinism or QTL* or SNP or polymorphism* or polymorphic or linkage or pigmentation or pigmented or knockout or KO or knock-out or congenic or karyotype or (chromosomal adj anomalies) or transgenic or ablated or ablation or genomes or cloned or (Wistar adj albino) or (H2 adj locus) or (chromosome adj substitution*) or (albino adj locus) or (agouti adj loci)))

**Animal Molecular Genetics:**

(((GWAS OR QTL OR (candidate adj1 gene) OR congenic OR transgenic OR X-linked OR (drosophila AND mutant) OR (drosophila AND mutation) OR DNA OR transpolygenic OR Y-linked OR mutant OR (gene adj1silencing) OR (Genome adj1 wide) or Genome-wide OR GWAS OR Genetic locus OR (Albino adj1 locus) OR (nucleotide adj1 polymorphism) OR (Chromosome AND allele) OR (Mitochondrial adj1 gene) OR (Genetical genomic) OR (Epigenetic AND gene expression) OR (Karyotype AND mice) OR (Drosophila AND polymorph*) OR (Cloned AND puppies) OR (Drosophila AND chromosom*) OR (Drosophila AND locus) OR (albino AND genetic variance) OR (genetic basis AND drosophila) OR (consomic rat) OR (drosophila AND genetic architecture) OR (loci AND hybrid) OR (albino AND inheritance) OR (Drosophila AND allele) OR (coisogenic AND albino) OR Albinism OR knock-out OR knock-out OR mutation OR QTLs OR (promoter adj1 polymorphism) OR Congenic OR CNV OR (Copy adj1 number adj1 variant) OR (Genetic adj1 mapping) OR (receptor adj1 gene) OR (Genomic adj1 imprinting) OR Lurcher mutant OR Lurcher mutation OR Gene mutation OR single-locus OR (inactive adj1 mutation) OR (single adj1 gene) OR (Histocompatibility complex AND genotype) OR (Drosophila AND mutation*) OR (histocompatibility complex AND gene) OR (transporter AND polymorphism) OR (SNP AND polymorphism) OR (genetic AND polymorphism) OR (Y chromosome AND linkage) OR (gene knockout) OR (locus AND genotype) OR (genus AND genetic architecture) OR (drosophila AND gene affects) OR (drosophila AND mutant*) OR (mice AND targeted disruption) OR (overexpressing mice) OR (chromosome AND mRNA) OR (chromosom* AND inbred) OR (receptor adj1 gene) OR (transporter adj1 gene) OR (Linkage adj1 map*) OR (Null adj1 mutant) OR (null adj1 allele)).ab,ti.) NOT ((exp cell signaling/) OR (exp neurotransmission/) OR (exp synapses/) OR (exp neural receptors/) OR coeruleus))
